# An Improved Method of General Anesthesia in Head-Surgery by Means of Glass Nasal Tubes

**Published:** 1910-11-15

**Authors:** Joseph E. Lumbard

**Affiliations:** New York


### AN IMPROVED METHOD OF GENERAL ANESTHESIA IN HEAD-SURGERY BY MEANS OF GLASS NASAL TUBES.

JOSEPH E. LUMBARD, M.D., NEW YORK.

Anesthesia for head-surgery certainly presents difficulties for the anesthetist. In order to insure greater safety to the patient and less interference of the anesthetist with the surgeon during an operation on the head, many inhalers have been produced. Nasal tubes for such purposes of anesthesia were introduced in 1908 by Dr. Edwin Pynchon of Chicago and Dr. Stuart B. Blakely of New York independently. Since one of these instruments allows the entrance of too much air and the other does not provide sufficient air, I have devised an instrument which experience has proved to be more practical than any other on the market.

My apparatus consists of two glass tubes so bent that each will properly fit a nostril. These tips are connected with the mechanism which supplies the anesthetic vapor by means of two soft rubber tubes joined together at the supply end by a glass Y, which also connects them with the supply tube itself. They are furnished in three different sizes in order to make the apparatus adapted to nostrils of different shapes.

Furthermore, the apparatus which I use for continuing the anesthesia is different from that employed by eitherDr. Pynchon or Dr. Blakely. I induce anesthesia in the usual way and then introduce the glass nasal tips into the nose. For convenience these may be held in position by means of a small piece of adhesive plaster placed across them transversely on the forehead. The anesthesia is then continued by supplying the vapor in one of four ways: first, by the Crile method, that is, by the use of a funnel and tube on the stretched gauze of which ether is dropped; second, by the one-bottle Junker apparatus, as used so commonly in

England for head-surgery; third, by the two-bottle modification of the Junker apparatus, as used by Dr. T. W. Brophy of Chicago for work on cleft palates; fourth, by the Gwathmey three-bottle modification of the Junker apparatus. By each of the Junker methods anesthesia vapor is pumped into the nostrils by means of suitable bulbs.

I much prefer the Gwathmey apparatus, as it allows the use of both ether and chloroform, either singly or combined in any proportion, with the greatest safety and ease.

The following are some of the advantages of the glass nasal tips over the long rubber tubes which have been in common use:

1. They are more safely and easily introduced into the nose.

2. They allow the anesthetic vapor on its way to the lungs to becomed warmed by the nose

3. Successful use of the instrument is in no way interfered with by a deformed nasal septum or exostosis.

4. They do not cause nasal hemorrhage.

5. They cannot become obstructed with blood and mucus.

6. They remain in position much better than rubber tubes.

7. They are easier to clean and sterilize.

A method very similar to mine has been successfully employed at Roosevelt Hospital in New York City, where it is now entirely used for head-surgery in place of rectal anesthesia.

The glass nasal tips, as above illustrated, are certainly a most excellent means of producing a safe and continuous anesthesia in head-surgery, not interfering with the surgeon. —*A. M. A. Journal*, October 8, 1910.

